# Steatosis Alters the Activity of Hepatocyte Membrane Transporters in Obese Rats

**DOI:** 10.3390/cells10102733

**Published:** 2021-10-13

**Authors:** Catherine M. Pastor, Valérie Vilgrain

**Affiliations:** 1 Department of Radiology, University Hospital of Geneva, 1205, Geneva, Switzerland; 2 Centre de Recherche sur L’inflammation, Inserm, U1149, CNRS, ERL8252, F-75006 Paris, France; valerie.vilgrain@aphp.fr; 3 Department of Radiology, Hôpital Beaujon, Hôpitaux Paris Nord Val de Seine (AP-HP), Université de Paris, 92110 Clichy, France

**Keywords:** hepatocyte membrane transporter, hepatocyte concentrations, transporter activity, liver imaging

## Abstract

Fat accumulation (steatosis) in ballooned hepatocytes alters the expression of membrane transporters in Zucker fatty (*fa/fa*) rats. The aim of the study was to quantify the functions of these transporters and their impact on hepatocyte concentrations using a clinical hepatobiliary contrast agent (Gadobenate dimeglumine, BOPTA) for liver imaging. In isolated and perfused rat livers, we quantified BOPTA accumulation and decay profiles in *fa/+* (normal) and *fa/fa* hepatocytes by placing a gamma counter over livers. Profiles of BOPTA accumulation and decay in hepatocytes were analysed with nonlinear regressions to characterise BOPTA influx and efflux across hepatocyte transporters. At the end of the accumulation period, BOPTA hepatocyte concentrations and influx clearances were not significantly different in *fa/+* and *fa/fa* livers. In contrast, bile clearance was significantly lower in fatty hepatocytes while efflux clearance back to sinusoids compensated the low efflux into canaliculi. The time when BOPTA cellular efflux impacts the accumulation profile of hepatocyte concentrations was slightly delayed (2 min) by steatosis, anticipating a delayed emptying of hepatocytes. The experimental model is useful for quantifying the functions of hepatocyte transporters in liver diseases.

## 1. Introduction

Nonalcoholic fatty liver disease (NAFLD) includes various dysfunctions such as increased intrahepatic triglyceride content (steatosis), inflammation, and fibrosis [1,2]. The transition from fatty livers to more severe disease (steatohepatitis) is triggered by inflammation. Cholestasis (or decreased bile flow) is a marker of severity. Significant progress has been made to decrease the burden of NAFLD, but the translation of experimental treatments to the human disease has consistently failed [3]. One of the reasons is that the mechanisms underlying the disease are numerous and complex. Nevertheless, these experimental models contribute to a better understanding of the disease.

**Figure 1:**
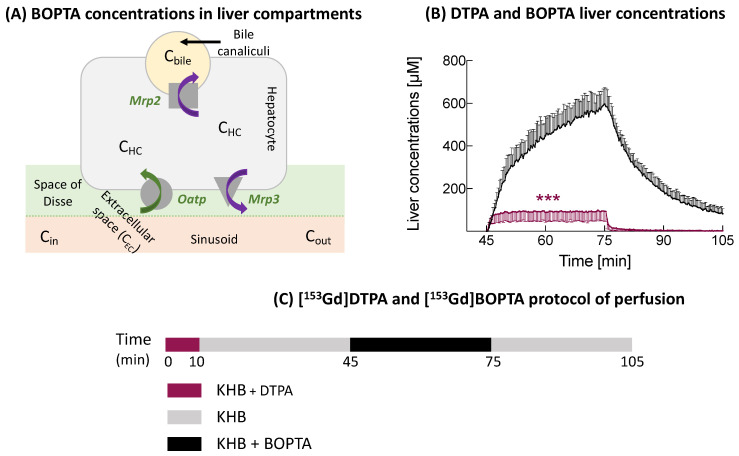
(**A**) Distribution of BOPTA concentrations in liver compartments: portal vein (C_in_), hepatic veins (C_out_), extracellular space (C_EC_), hepatocytes (C_HC_), and bile canaliculi (C_bile_). (**B**) Liver concentrations of contrast agents are measured by a gamma counter placed over livers. DTPA preperfusion was used to measure C_EC_ (purple symbols). DTPA distributes only into the extracellular space. DTPA concentrations were reported with BOPTA liver concentrations (***). BOPTA (black symbols) distributes into the extracellular space, hepatocytes, and bile canaliculi. (**C**) [^153^Gd]DTPA and [^153^Gd]BOPTA were diluted in Krebs–Henseleit bicarbonate (KHB) solution to obtain a 200-µM concentration. Livers were successively perfused with 200-µM [^153^Gd]DTPA (10 min), KHB solution (35 min), 200-µM [^153^Gd]BOPTA (30 min, accumulation or perfusion period), and KHB solution (30 min, decay or elimination period).

Thus, NAFLD modifies the expression of transporters located on the basolateral and canalicular membranes of hepatocytes. Human Organic Anion Transporting Polypeptide B1/B3 (OATP1B1/B3) are not altered in steatosis, but their expression is decreased in patients with steatohepatitis [4]. In contrast, the Multidrug Resistance Associated Protein 3 (MRP3) expression is increased in fatty livers and steatohepatitis. MRP2 is upregulated in steatohepatitis [5]. The activity of these transporters determines the hepatocyte concentrations of endogenous substrates and drugs that might be unpredictable in fatty livers. Thus, patients with steatohepatitis have higher liver concentrations of mebrofenin (MEB, Choletec^®^, Bracco imaging, Milan, Italy) than volunteers [6]. MEB is transported across hepatocytes via human OATPs, MRP2, and MRP3 (Figure 1A).

Experimental nonalcoholic fatty livers can be investigated in Zucker fatty (*fa/fa*) rats [7,8]. These rats have a mutated leptin receptor and a decreased leptin affinity. Rats develop severe obesity with insulin resistance and hepatic steatosis. The spontaneous progression of steatosis to steatohepatitis is rare and needs a second hit [9]. Heterozygous *fa/+* rats serve as control animals. In biopsies, we showed previously that hepatocytes in *fa/fa* livers are distended by large fat droplets [10]. This fat accumulation increases the hepatocyte volume and total liver weights [11]. No inflammation or fibrosis is detected. Steatosis is associated with high triglyceride and cholesterol serum concentrations [10]. Portal pressures remain normal, but bile flow rates were significantly decreased in comparison to normal livers.

The expression of hepatocyte transporters was also investigated in *fa/fa* livers. According to Canet et al. [12], Oatp1a4 expression is downregulated while that of Oatp1b2 is induced in *fa/fa* livers. In contrast, Mrp2 and Mrp3 expression is not significantly different in *fa/fa* and control Sprague-Dawley livers. Another experimental study found that rats fed with a high fat diet had a decreased mRNA expression of *Slco1a2*, *Slco1b2*, *Abcc2*, and *Abcc3* (genes coding for Oatps, Mrp2, and Mrp3) [13]. However, the protein expression of the transporters was not available.

We previously showed that *fa/fa* rats have a decreased bile excretion of Gadobenate dimeglumine (BOPTA, Multihance^®^, Bracco Imaging, Milan, Italy) [10]. BOPTA is a clinical hepatobiliary contrast agent that characterises liver parenchyma and focal lesions [14]. BOPTA distributes into the extracellular compartment and enters into rat hepatocytes by the Organic anion transporting polypeptide transporters (Oatp1a1, Oatp1a4, and Oatp1b2) (Figure 1A) [15]. BOPTA is excreted unchanged (no metabolism) into bile canaliculi through the Multidrug resistance-associated protein 2 (Mrp2) [16]. BOPTA can also return back to sinusoids using Mrp3. BOPTA accumulation in hepatocytes is modulated by the activity of these influx and efflux transporters.

In an ex-vivo model of isolated and perfused rat liver (IPRL), we previously published the distribution of BOPTA in the extracellular space, hepatocytes, and bile canaliculi [17]. By placing a gamma counter over livers, it is possible to measure the liver concentrations of labelled BOPTA (Figure 1B). A preperfusion of labelled gadopentetate dimeglumine (DTPA) quantifies the extracellular concentrations, because it distributes exclusively into the extracellular space. Concentrations of bile canaliculi and hepatocytes were calculated. We found that the maximal BOPTA hepatocyte concentrations (obtained at the end of the perfusion period) were similar in *fa/+* and *fa/fa* livers. A compartmental pharmacokinetic modelling determined that the BOPTA influx rates were similar in both groups while the efflux rates from hepatocytes into bile and back into sinusoids were decreased.

In the present study, we reanalysed the row data of *fa/+* and *fa/fa* livers to quantify the BOPTA compartmental distribution (or respective contribution of the extracellular space, hepatocytes, and bile canaliculi to liver concentrations) and new pharmacokinetic parameters that characterise BOPTA accumulation and decay in hepatocytes.

## 2. Materials and Methods

### 2.1. Isolated and Perfused Rat Livers

Before liver isolation, Zucker fat (*fa/fa*, *n* = 5) and Zucker lean (*fa/+*, *n* = 5) rats were anesthetised with pentobarbital (50 mg · kg^−1^, ip). In these two groups, we published previous biological parameters of liver functions while the transporter functions were assessed by pharmacokinetic modelling [10]. In the present study, we returned to row data to analyse the new hepatic pharmacokinetic parameters. Rat livers were isolated, leaving the organs in the carcass. The abdominal cavity was opened and the portal vein cannulated. The hepatic artery was not perfused. The abdominal vena cava was transected, and an oxygenated Krebs–Henseleit bicarbonate (KHB) solution was pumped into the portal vein, the solution being discarded after liver distribution via a vena cava transection. The flow rate was slowly increased over 1 min up to 30 mL/min. In the second step, the chest was opened, and a cannula was inserted through the right atrium to collect solutions flowing from the hepatic veins. Finally, the abdominal inferior vena cava was ligated, allowing solutions perfused by the portal vein to be eliminated by the hepatic veins.

The perfusion system included a reservoir, a pump, a heating circulator, a bubble trap, a filter, and an oxygenator. Solutions of perfusion were equilibrated with a mixture of 95% O_2_ and 5% CO_2_. Livers were continuously perfused with fresh solutions using a nonrecirculating system. The common bile duct was cannulated with a PE_10_ catheter, and bile samples were collected every 5 min to measure the bile flow rates (Q_bile_, µL/min/liver) and BOPTA concentrations (C_bile_, µM). Samples were collected from hepatic veins every 5 min (C_out_, µM). C_out_ during the BOPTA perfusion period were concentrations that did not enter into hepatocytes plus concentrations that entered into hepatocytes (via Oatps) and returned into sinusoids via Mrp3 (Figure 1A). During the rinse period, C_out_ were concentrations leaving hepatocytes because the concentration in the portal vein (C_in_) was 0.

An adequate viability of livers was assessed by a steady portal pressure below 12 mmHg during the entire protocol. We previously published that a flow rate of 30 mL/min/liver maintains a normal liver O_2_ consumption [18].

### 2.2. Perfusion of DTPA and BOPTA

Rat livers were perfused with gadopentetate dimeglumine (DTPA; Magnevist^®^; Bayer imaging, Berlin, Germany) and gadobenate dimeglumine (BOPTA, MultiHance^®^; Bracco Imaging, Milan, Italy). DTPA distributes only within sinusoids and the interstitium, while BOPTA distributes into the extracellular space, hepatocytes, and bile canaliculi. DTPA and BOPTA labelled with ^153^Gd were obtained by adding ^153^GdCl_3_ (1 MBq/mL) to the commercially available (0.5 M) solutions of DTPA and BOPTA. Then, [^153^Gd]DTPA and [^153^Gd]BOPTA were diluted in the KHB solution to obtain a 200-µM concentration. Livers were successively perfused with 200 µM [^153^Gd]DTPA (10 min), KHB solution (35 min), 200 µM [^153^Gd]BOPTA (30 min, accumulation or perfusion period), and KHB solution (30 min, decay or elimination period) (Figure 1C). The protocol lasted 105 min for each group.

### 2.3. Concentrations in Liver Compartments

To quantify the BOPTA concentrations in liver compartments, a gamma counter that collects count rates every 20 s was placed 1 cm above a right liver lobe. The counter measured the radioactivity in a region of interest that was identical in each liver. To transform count rates into BOPTA concentrations, the total liver radioactivity was measured by an activimetre at the end of each experiment and related to the last count rates. Radioactivity was corrected for decay. The gamma counter delineated a region of interest in a liver lobe from which all count rates originating from the extracellular space, hepatocytes, and bile canaliculi were divided by the liver weight to obtain liver concentrations (C_liver_, µM). To calculate the hepatocyte concentrations, we must eliminate the BOPTA concentrations located inside the extracellular space (C_EC_) and bile canaliculi (C_BC_). BOPTA extracellular concentrations cannot be measured, because the substrate rapidly enters into hepatocytes (<2 min). We used DTPA that distributes only in the extracellular space to estimate the BOPTA C_EC_. Assuming that DTPA C_EC_ is similar to BOPTA C_EC_, we can subtract BOPTA C_liver_ − DTPA C_liver_ (or DTPA C_EC_). The concentrations were constant during the 10-min perfusion. During BOPTA perfusion, this plateau was extended over 30 min (Figure 1B). We assumed that the concentrations inside bile canaliculi were similar to those measured in the common bile duct (C_bile_, µM), although solute export from cholangiocytes and water transport along ductules and ducts may modify the primary bile in canaliculi. The volume ratio of the bile canaliculi and liver was previously estimated by Blouin et al. [19] at 0.43%. Concentrations in the bile canaliculi detected by the counter (C_BC_) were then equal to 0.0043 · C_bile_. Hepatocyte concentrations in a 78% liver volume (C_HC78%_) detected by the counter were C_liver_ − C_EC_ − 0.0043 C_bile_. Indeed, Blouin et al. [19] determined previously that the volume ratio of hepatocytes to liver in the absence of fibrosis or inflammation was 78%. In situ C_HC100%_ were calculated by (100/78) · C_HC78%_.

### 2.4. Transfer Rates and Clearances between Compartments

The BOPTA removal rate from sinusoids during the perfusion period (v, nmol/min) was measured by Q_H_ · (C_in_ − C_out_), where Q_H_ was the constant liver flow rate (30 mL/min), C_in_ (µM) was the constant portal concentration, and C_out_ (µM) was the concentration measured in hepatic veins. The unbound fraction in the solutions was 1, because no protein was added into the solutions. Hepatic clearance (CL_H_, mL/min) was the ratio of v and C_in_ during the last min of perfusion. The BOPTA extraction ratio (ER) was (C_in_ − C_out_)/C_in_. The BOPTA biliary excretion rate (*v*_bile_, nmol/min) was C_bile_ · Q_bile_, where C_bile_ (µM) was the concentration in the common bile duct, and Q_bile_ was the bile flow rate (µL/min/liver weight). Clearance from the hepatocytes to bile canaliculi (CL_bile_, mL/min) was the slope of the linear regression between *v*_bile_ (*Y*-axis) and the hepatocyte concentrations (C_HC_, *X*-axis). CL_bile_ was measured during the entire protocol. During the rinse period, BOPTA concentrations leaving hepatocytes into sinusoids (C_ef_, µM) were measured by C_out_ in the absence of BOPTA entry into livers. The basolateral efflux from hepatocytes into sinusoids (*v*_ef_, nmol/min) was C_ef_ · Q_H_, and the basolateral clearance (CL_ef_, mL/min) was the slope of linear regression between *v*_ef_ (*Y*-axis) and C_HC_ (*X*-axis). With the assumption that CL_ef_ was similar during both the perfusion and rinse periods, we estimated C_ef_ during the accumulation period by (C_HC_ · CL_ef_)/Q_H_. The hepatocyte influx rate *v*_in_ (nmol/min) was [C_in_ − (C_out_ − C_ef_)] · Q_H_, and the hepatocyte influx clearance CL_in_ (mL/min) was *v*_in_/C_in_. The CL_in_ was measured during the last min of perfusion.

### 2.5. Accumulation Profile of Hepatocyte Concentrations

During the perfusion period, BOPTA hepatocyte accumulation was best described by a segmental linear regression obtained from GraphPad Prism version 8, GraphPad Software, La Jolla, CA, USA [20]. This function defines a first line L_1_ for a time below T_0_ and a second line L_2_ for a time higher than T_0_. T_0_ is the time when the two lines would intersect. No constraint was applied to fit the data.

### 2.6. Concentration Ratios (R) between Compartments

In in vivo studies, only liver-to-plasma concentration ratios are available. However, concentrations facing the sinusoidal membrane of hepatocytes where Oatps reside are the extracellular concentrations, because the space of Disse intertwines between sinusoids and the sinusoidal membrane. Thus, the hepatocyte-to-extracellular concentration ratio (R_HC/EC_) characterises BOPTA transport across Oatps. R_HC/EC_ was measured at T_0_ before the BOPTA efflux out of cells. The bile-to-hepatocyte concentration ratio (R_bile/HC_) was the slope of the relationship between C_bile_ (*Y*-axis) and C_HC_ (*X*-axis) during the entire protocol. R_bile/HC_ was independent of the BOPTA influx into hepatocytes. The hepatic vein-to-hepatocyte ratio (R_HV/HC_) was the slope of the relationship between C_out_ (*Y*-axis) and C_HC_ (*X*-axis) during the rinse period. R_HV/HC_ was independent of the BOPTA influx into the hepatocytes. The concentrations used in these ratios were in situ concentrations. Finally, the bile-to-extracellular concentration ratio (R_bile/EC_) assessed the ability of transporters to concentrate BOPTA from the extracellular space to bile compartments. R_bile/EC_ was measured at the end of the perfusion period, when the bile concentration was maximal.

### 2.7. Decay Profile of Hepatocyte Concentrations

During the decay period, the data were best described by a one phase decay (GraphPad Prism version 8, GraphPad Software, La Jolla, CA, USA) [21]. The plateau was constrained to 0, because BOPTA must leave hepatocytes. The model was defined by a rate constant of elimination (k_el,HC_, min^−1^). Knowing CL_bile+ef_ and k_el,HC_, we calculated the apparent hepatocyte volumes (V_HC_, mL) as CL_bile+ef_/k_el,HC_.

### 2.8. Statistics

Data were the means ± SD. The parameters obtained in normal and fatty livers were compared with a Mann–Whitney test (GraphPad Prism version 8, GraphPad Software, La Jolla, CA, USA).

## 3. Results

### 3.1. Basic Pharmacokinetic Parameters

**Figure 2:**
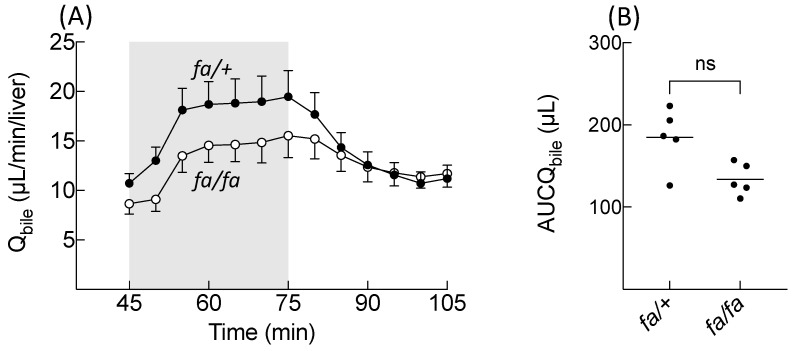
(**A**) Bile flow rates (Q_bile_) during the BOPTA perfusion and rinse periods. Livers were perfused with Krebs–Henseleit bicarbonate solution (KHB) + 200 µM [^153^Gd]BOPTA (45–75 min, grey area) and KHB (75–105 min). (**B**) Area under the curve of the Q_bile_ increase from 45 to 75 min. Control livers (*fa/+*, black circles) and fatty livers (*fa/fa*, open circles).

The fatty livers were cholestatic before BOPTA perfusion. The bile flow rates (Q_bile_) were 10.7 ± 1.0 (*fa*/+ livers) and 8.7 ± 1.0 µL/min/liver (*fa/fa* livers, *p* = 0.03, Figure 2A, before BOPTA perfusion, time point: 45 min). Then, BOPTA perfusion increased bile flow rates during the perfusion period (45–75 min). The area under the curve of the bile increase (AUC_Qbile_) was not significantly different in fatty (134 ± 19 µL) and normal (185 ± 36 µL) livers (*p* = 0.06, Figure 2B). At the end of the perfusion period, the BOPTA liver extraction ratios were low and not significantly different in the two groups (6 ± 1% in *fa*/+ livers and 5 ± 1% in *fa/fa* livers, *p* = 0.17).

### 3.2. Accumulation in Liver Compartments

**Figure 3:**
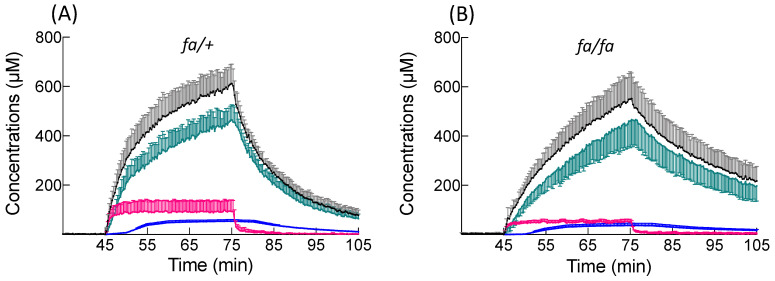
BOPTA compartmental distribution in *fa*/+ (**A**) and *fa/fa* (**B**) livers. Livers were perfused with Krebs–Henseleit bicarbonate solution (KHB) + 200 µM [^153^Gd]BOPTA (45–75 min) and KHB alone (75–105 min). Liver concentrations (black symbols) were measured by a gamma counter. Concentrations in the extracellular compartment (red symbols) were measured during the previous DTPA perfusion. Concentrations that originated from bile canaliculi (blue symbols) and from 78% hepatocytes (green symbols) were calculated.

BOPTA accumulated in three compartments during the perfusion period (Figure 3 and Table 1). In the extracellular compartment, the concentrations (C_EC_) were steady and not significantly different in the two groups. C_EC_ contributed to 15 ± 7% (*fa/+* livers) and 10 ± 3% (*fa/fa* livers) of the liver concentrations (*p* = 0.22). The maximal hepatocyte concentrations detected by the counter (C_HC78%_) were also similar in lean (465 ± 61 µM) and fatty (461 ± 109 µM) livers (*p* = 0.70). In *fa/+* livers, the BOPTA maximal concentrations detected by the counter in bile canaliculi (C_BC_) were 54 ± 7 µM and accounted for 9 ± 1% of the liver concentrations. In *fa/fa* livers, BOPTA C_BC_ was 37 ± 9 µM (*p* = 0.02 vs. *fa/+* livers) and accounted for 7 ± 2% of C_liver_ (*p* = 0.10 vs. *fa/+* livers). BOPTA C_out_ in hepatic veins were slightly lower than the portal vein concentrations C_in_, reflecting the low liver extraction ratio of BOPTA. C_out_ were not significantly different between the two groups. At the end of the accumulation period, BOPTA compartmental distribution was similar in the extracellular space and hepatocytes for both groups. However, the accumulation inside bile canaliculi was significantly lower in cholestatic livers. Nevertheless, the liver concentrations were not significant different between the two groups.

### 3.3. Accumulation Profile of BOPTA Concentrations in Hepatocytes

**Figure 4:**
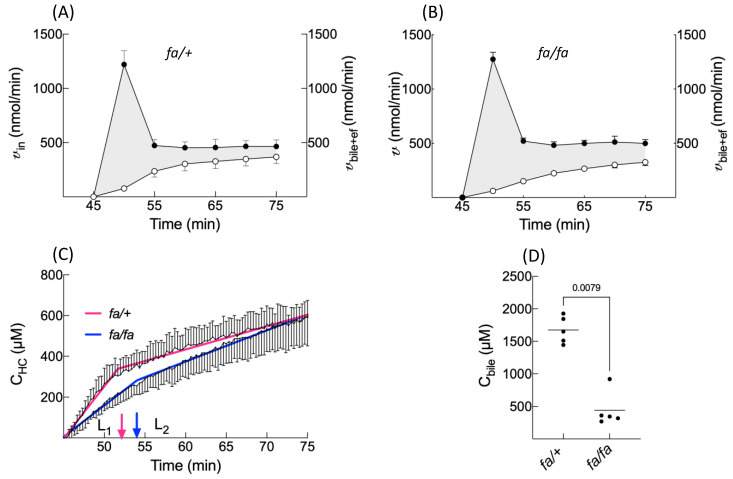
BOPTA hepatocyte influx rates (*v*_in_, left *Y*-axis, black circles) and bile plus basolateral efflux rates (v_bile+ef_, right *Y*-axis, open circles) during the BOPTA accumulation period from 45 to 75 min in *fa/+* (**A**) and *fa/fa* (**B**) livers. Livers (*n* = 10) were perfused with Krebs–Henseleit bicarbonate solution (KHB) + 200 µM [^153^Gd]BOPTA. Difference between *v*_in_ and *v*_bile+ef_ (grey area). (**C**) Accumulation of BOPTA hepatocyte concentrations from 45 to 75 min. Accumulation was best described by a segmental linear regression (red curve for *fa/+* and blue curve for *fa/fa*). T_0_ (red and blue arrows) were the times when BOPTA cellular efflux impacted the hepatocyte accumulation. (**D**). Bile concentrations measured 5 min after the start of BOPTA perfusion (50 min of the experimental protocol).

The accumulation profile of BOPTA concentrations in hepatocytes relied on the difference between the influx rates (*v*_in_, nmol/min) and efflux rates (*v*_bile+ef_, nmol/min) (Figure 4A,B, grey areas and Table 2). In both groups, *v*_in_ remained higher than *v*_bile+vef_. At the end of the accumulation period, *v*_bile+ef_ was not significantly different between the two groups. However, *v*_bile_ was significantly lower (*p* = 0.02) and *v*_ef_ (*p* = 0.06) was higher in *fa/fa* than *fa/+* livers (Table 2). Thus, in fatty livers, BOPTA efflux back into sinusoids compensates for the decreased bile excretion.

BOPTA hepatocyte accumulation was best described by a segmental linear regression (Figure 4C) [20]. This function defined a first line L_1_ for a time below T_0_ and a second line L_2_ for a time higher than T_0_, while ensuring that both lines intersected at T_0_. T_0_ was the time when BOPTA efflux from hepatocytes interfered with hepatocyte accumulation. T_0_ occurred 7 ± 1 min (*fa/+* livers) and 9 ± 2 min (*fa/fa* livers) after the start of BOPTA perfusion (*p* = 0.02). The L_1_ slopes were 52 ± 16 µM/min (*fa/+* livers) and 31 ± 4 µM/min (*fa/fa* livers, *p* = 0.01). These slopes characterised BOPTA influx into hepatocytes by Oatps. After T_0_, the L_2_ slopes were lower than the L_1_ slopes (*fa/+* livers: 11 ± 1 µM/min and *fa/fa* livers: 15 ± 4 µM/min, *p* = 0.06). Concomitant entry and efflux from the hepatocytes explained this decrease. Five minutes after the start of BOPTA perfusion, the bile concentrations were 1676 ± 208 (*fa/+* livers) and 443 ± 269 (*fa/fa* livers) (*p* = 0.01, Figure 4D). In comparison to the lean livers, the fatty livers had a delayed T_0_, decreased L_1_ slopes (hepatocyte influx), similar L_2_ slopes, and decreased bile concentrations.

At the end of the accumulation period, the BOPTA influx clearance (CL_in_) was 2.3 ± 0.3 mL/min (*fa/+* rats) and 2.5 ± 0.2 mL/min (*fa/fa* rats, *p* = 0.55, Table 2). CL_H_ defined by (C_in_ − C_out_) · Q_H_ was lower than CL_in_, which was defined by [C_in_ − (C_out_ − C_ef_)] · Q_H_). CL_H_ and CL_in_ were not significantly different in the two groups.

### 3.4. Concentration Ratios (R) between Compartments

The concentration ratio between the hepatocytes and extracellular space (R_HC/EC_) at T_0_ was not significantly different between groups (Table 3). R_HC/EC_ was measured before BOPTA hepatocyte efflux. The concentration ratio between the bile canaliculi and hepatocytes (R_bile/HC_) was the slope of relationship between C_HC_ (*X*-axis) and C_bile_ (*Y*-axis) during the perfusion and rinse periods. R_bile/HC_ was not significantly different between groups. In contrast, R_HV/HC_ was significant higher in fatty livers, and more BOPTA returned to sinusoids. R_HV/HC_ was the slope of the relationship between C_HC_ (*X*-axis) and C_out_ (*Y*-axis) during the rinse period (no BOPTA entry into livers). Finally, the maximal concentration ratio between the bile and extracellular space (R_bile/EC_) quantified the ability of transporters to concentrate BOPTA from the extracellular space to bile. The ratio was measured at the end of the perfusion period and was not significantly different between groups.

### 3.5. BOPTA Elimination from Hepatocytes

**Figure 5:**
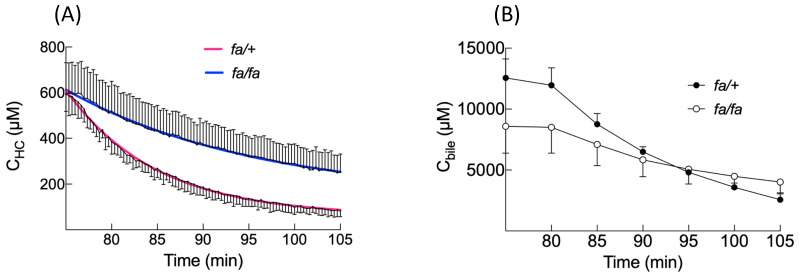
BOPTA hepatocyte (**A**) and bile (**B**) concentrations during the rinse period (75–105 min) when the livers were perfused only with the Krebs–Henseleit bicarbonate solution. Hepatocyte decay was best described by a one phase decay (red curve for *fa/+* and blue curve for *fa/fa*).

**Figure 6:**
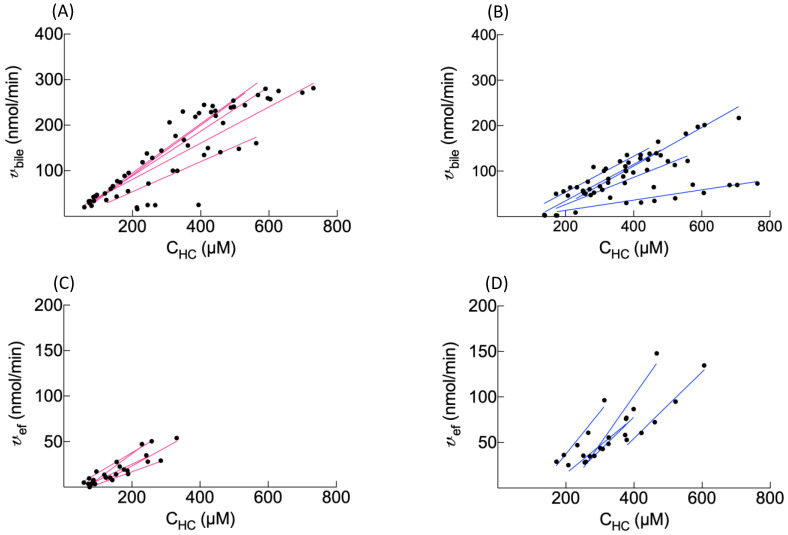
BOPTA clearances from the hepatocytes to bile canaliculi (CL_bile_) were determined by the slope of linear regression between *v*_bile_ (*Y*-axis) and C_HC_ (*X*-axis) in *fa/+* (**A**) and *fa/fa* (**B**) livers. The BOPTA basolateral efflux clearance (CL_ef_) was measured during the rinse period by the slope of linear regression between *v*_ef_ (*Y*-axis) and C_HC_ (*X*-axis) in *fa/+* (**C**) and *fa/fa* (**D**) livers. Linear regressions in normal (**A** and **C**, red) and fatty (**B** and **D**, blue) livers.

We used two ways to assess the BOPTA efflux from hepatocytes: BOPTA hepatocyte concentration decay and BOPTA recovery in sinusoids (CL_ef_) plus bile canaliculi (CL_bile_). During the rinse period, hepatocyte concentration decay was best described by a one phase decay (Figure 5A and Table 4). The model was described by a rate constant of elimination (k_el,HC_) that was significantly decreased in fatty livers (Table 4). CL_bile_ and CL_ef_ were defined by the linear regression between *v*_bile_ or *v*_ef_ (*Y*-axis) and the hepatocyte concentrations (C_HC,_ *X*-axis) (Figure 6). CL_bile_ was significantly lower in *fa/fa* livers (0.26 ± 0.11 mL_HC_/min) than in *fa/+* livers (0.46 ± 0.09 mL_HC_/min, *p* = 0.02, Table 2). In contrast, CL_ef_ was significantly higher in *fa/fa* livers (0.41 ± 0.09 mL_HC_/min) than in *fa/+* livers (0.20 ± 0.05 mL_HC_/min, *p* = 0.01). In fatty livers, high CL_ef_ compensated the low CL_bile_, and CL_bile+ef_ was similar in both groups (Table 2). The ratios between CL_ef_ and CL_bile+ef_ were 62 ± 13% (*fa/fa* livers) and 31 ± 7% (*fa/+* livers) (*p* = 0.01). Knowing CL_bile+ef_ and k_el,HC_, we calculated the apparent hepatocyte volume (V_HC_, ml) as CL_bile+ef_/k_el,HC_. The V_HC_ were significantly higher in *fa/fa* livers.

## 4. Discussion

BOPTA is a clinical hepatobiliary contrast agent that characterises liver disease and focal lesions in liver magnetic resonance imaging [14]. BOPTA distributes within the extracellular compartment and enters into rat hepatocytes by Organic anion transporting polypeptide transporters (Oatp1a1, Oatp1a4, and Oatp1b2) [15]. The contrast agent is excreted unchanged (no metabolism) into bile canaliculi through Mrp2 [16]. BOPTA can also return back to sinusoids using Mrp3. BOPTA hepatocyte accumulation is modulated by the activity of the three transporters. In this study, we showed that BOPTA has a low liver extraction ratio (<7%) that is not modified by steatosis. BOPTA transport by Mrp2 is associated with water transport (choleresis). We showed that a BOPTA-induced bile flow increase is not significantly different in *fa/fa* and *fa/+* livers. However, a single low value of AUCQ_bile_ in the *fa/+* group might hide the statistical difference between the groups. The water transport across the canalicular membrane may be linked to BOPTA crossing via Mrp2, which triggers the trafficking and insertion of aquaporin-8-containing vesicles into the canalicular membrane [22,23]. BOPTA-induced choleresis is concentration-dependent and inhibited in livers lacking Mrp2 [16]. Similar results were published with benzylpenicillin [24].

BOPTA distributes into the extracellular space before reaching Oatps. In accordance with the absence of fibrosis in fatty livers [10], steatosis does not modify the extracellular concentrations that represent 13% of the liver concentrations in both groups. The maximal hepatocyte concentrations are also similar, as are the liver concentrations. Consequently, liver imaging following BOPTA injection would not detect differences between the two groups. However, BOPTA accumulation profiles are modified in steatosis. BOPTA accumulation into hepatocytes is best described by segmental linear regression [20]. This function defines a first line L_1_ for a time before T_0_ (before BOPTA efflux from hepatocytes). T_0_ is slightly delayed in fatty hepatocytes, anticipating a delay in cellular emptying. Steatosis decreases the L_1_ slope or BOPTA entry into fatty hepatocytes. After T_0_, L_2_ slopes are much lower than L_1_ slopes, because BOPTA efflux from hepatocytes counteracts the influx. Steatosis does not modify the L_2_ slopes.

Another parameter of BOPTA influx into hepatocytes is the concentration ratio between the hepatocytes and extracellular space (R_HC/EC_) measured at T_0_ before BOPTA cellular efflux. Steatosis does not modify this ratio. BOPTA influx clearance (CL_in_) and hepatic clearance (CL_H_) are also similar at the end of BOPTA perfusion, confirming that steatosis does not substantially modify the Oatp activity. Geier et al. [11] found a decreased expression of Oatp1a4 but not Oatp1a1 in *fa/fa* livers. The transport activities were not investigated in this study. The reason why the L_1_ slope is lower in *fa/fa* than *fa/+* hepatocytes might be explained by the larger volume of fatty hepatocytes, the L_1_ slope being expressed in µM/min or nmol/(mL · min). With similar v_in_ in both groups, the L_1_ slope will be lower in a larger volume of fatty hepatocytes.

The BOPTA accumulation in bile (C_bile_) is significantly lower during the accumulation period in *fa/fa* livers, and the C_bile_ decrease during the rinse period is slower in fatty livers. Accordingly, the bile clearance (CL_bile_) is lower in fatty livers, showing a decreased activity of Mrp2 associated with steatosis. The low activity of Mrp2 can be attributed to a lower expression [11,25]. In contrast, efflux clearance back into sinusoids (CL_ef_) is significantly higher in fatty livers, reflecting an increased activity of Mrp3 that compensates the decreased activity of Mrp2. Thus, the sum of both efflux clearances (CL_bile+ef_) is not significantly different between both groups. Such compensation was previously published [26]. Nevertheless, the Mrp3 expression is not increased in *fa/fa* livers [11,25], suggesting that a dysregulation might promote BOPTA transport without changing the transporter expression. In contrast, the human MRP3 expression is increased in fatty livers and steatohepatitis [4]. Another way to describe BOPTA hepatocyte emptying is to compare the rate constant of elimination (k_el,HC_) obtained with the one phase decay equation. The k_el,HC_ that quantifies BOPTA efflux into bile canaliculi and sinusoids is lower in the presence of steatosis. Knowing CL_bile+ef_ and k_el,HC_, we can calculate the apparent hepatocyte volume where BOPTA distributes (V_HC_). The V_HC_ is significantly higher in *fa/fa* livers. However, these apparent hepatocyte volumes overestimate the hepatocyte volume calculated by 0.78 · liver weights.

When we previously determined the transporter activities of Oatps, Mrp2, and Mrp3 by pharmacokinetic modelling, we found that the BOPTA hepatocyte influx in *fa/fa* rats was not significantly different from *fa/+* livers [10]. In contrast, the bile and sinusoidal effluxes were both significantly decreased. The pharmacokinetic modelling did not evidence that a high efflux into sinusoids can compensate for a decreased efflux into bile canaliculi. The direct quantification of transporter activities we proposed in the present study is innovative and might validate new modelling approaches. The experimental model is useful to quantify the functions of hepatocyte transporters in liver diseases.

The isolated and perfused rat liver is a convenient model, because the experimental conditions are well-controlled and simplified. The structure of the liver is maintained. However, livers are perfused only through the portal vein, avoiding the complexity of a dual-input entry but changing the physiology of liver perfusion. To simplify the protocol, we did not add proteins in the KHB solution, and BOPTA was free to enter into hepatocytes. The gamma counter placed over the liver detects the concentrations of imaging substrates every 20 s, avoiding the collection of serial biopsy samples that damage rat livers. An important assumption is that the region of interest measured by the counter is representative of the entire liver. Moreover, the hepatocyte concentrations we obtained averaged out the concentrations of numerous hepatocytes, knowing that Oatps are mainly expressed in perivenous hepatocytes where BOPTA is likely to enter. Additionally, extrapolation of the results to other species and humans must be cautious, because the expressions of membrane transporters can differ.

## Figures and Tables

**Table 1:** BOPTA concentrations at the end of the accumulation period.

Livers	*fa/+*	*fa/fa*	*p*
C_in_ (µM)	200	200	
C_out_ (µM)	189 ± 2	190 ± 1	0.19
C_EC_ (µM)	93 ± 47	52 ± 10	0.22
C_BC_ (µM)	54 ± 7	37 ± 9	0.02
C_HC78%_ (µM)	465 ± 61	461 ± 109	0.70
C_bile_ (µM)	12564 ± 1550	8594 ± 2208	0.02
C_liver_ (µM)	612 ± 79	550 ± 110	0.31

**Table 2:** BOPTA transfer rates and clearances at the end of the accumulation period.

Livers	*fa/+*	*fa/fa*	*p*
*v* (nmol/min)	342 ± 66	291 ± 21	0.17
*v*_in_ (nmol/min)	465 ± 60	500 ± 35	0.29
*v*_bile_ (nmol/min)	246 ± 50	117 ± 30	0.02
*v*_ef_ (nmol/min)	122 ± 33	209 ± 54	0.06
*v*_bile+ef_ (nmol/min)	368 ± 60	326 ± 31	0.19
CL_H_ (ml_KHB_/min)	1.7 ± 0.3	1.5 ± 0.1	0.19
CL_in_ (ml_KHB_/min)	2.3 ± 0.3	2.5 ± 0.2	0.55
CL_bile_ (ml_HC_/min)	0.46 ± 0.09	0.26 ± 0.11	0.02
CL_ef_ (ml_HC_/min)	0.20 ± 0.05	0.41 ± 0.09	0.01
CL_bile+ef_ (ml_HC_/min)	0.66 ± 0.11	0.67 ± 0.17	1.00

**Table 3:** BOPTA concentration ratios between liver compartments.

Livers	*fa/+*	*fa/fa*	*p*
R_HC/EC_	4.1 ± 1.8	5.5 ± 1.3	0.15
R_bile/HC_	20.2 ± 3.2	18.6 ± 5.8	0.84
R_HV/HC_	0.0068 ± 0.0018	0.0135 ± 0.0030	0.01
R_bile/EC_	162 ± 69	109 ± 54	0.15

**Table 4:** Parameters of BOPTA hepatocyte decay.

Livers	*fa/+*	*fa/fa*	*p*
Y_0_ (µM)	587 ± 81	598 ± 137	1.00
k_el,HC_ (min^−1^)	0.08 ± 0.02	0.03 ± 0.01	0.01
T_1/2_ (min)	9 ± 2	23 ± 4	0.01
V_HC_ (ml)	9 ± 2	21 ± 2	0.01
78% of liver weight (mL)	7 ± 1	11 ± 2	0.01

Hepatocyte concentrations at the beginning of the rinse period (Y_0_). Decay rate constant (k_el,HC_). Time when Y equals Y_0_/2 (T_1/2_). Apparent hepatocyte volume (V_HC_).

## Data Availability

The data presented in this study are available on request from the corresponding author.

## References

[1] Fabbrini E., Sullivan S., Klein S. (2010). Obesity and nonalcoholic fatty liver disease: Biochemical, metabolic, and clinical implications. Hepatology.

[2] Geier A., Tiniakos D., Denk H., Trauner M. (2021). From the origin of NASH to the future of metabolic fatty liver disease. Gut.

[3] Tarantino G., Citro V., Capone D. (2019). Nonalcoholic fatty liver disease: A challenge from mechanisms to therapy. J. Clin. Med..

[4] Vildhede A., Kimoto E., Pelis R.M., Rodrigues A.D., Varma M.V.S. (2020). Quantitative proteomics and mechanistic modeling of transporter-mediated disposition in nonalcoholic fatty liver disease. Clin. Pharmacol. Ther..

[5] Dzierlenga A.L., Cherrington N.J. (2018). Misregulation of membrane trafficking processes in human nonalcoholic steatohepatitis. J. Biochem. Mol. Toxicol..

[6] Ali I., Slizgi J.R., Kaullen J.D., Ivanovic M., Niemi M., Stewart P.W., Barritt A.S., Brouwer K.L.R. (2018). Transporter-mediated alterations in patients with NASH increase systemic and hepatic exposure to an OATP and MRP2 substrate. Clin. Pharmacol. Ther..

[7] Soret P.A., Magusto J., Housset C., Gautheron J. (2020). In vitro and in vivo models of non-alcoholic fatty liver disease: A critical appraisal. J. Clin. Med..

[8] Sanches S.C., Ramalho L.N., Augusto M.J., da Silva D.M., Ramalho F.S. (2015). Nonalcoholic steatohepatitis: A search for factual animal models. Biomed. Res. Int..

[9] Kucera O., Cervinkova Z. (2014). Experimental models of non-alcoholic fatty liver disease in rats. World J. Gastroenterol..

[10] Pastor C.M., Wissmeyer M., Millet P. (2013). Concentrations of Gd-BOPTA in cholestatic fatty rat livers: Role of transport functions through membrane proteins. Contrast Media Mol. Imaging.

[11] Geier A., Dietrich C.G., Grote T., Beuers U., Prüfer T., Fraunberger P., Matern S., Gartung C., Gerbes A.L., Bilzer M. (2005). Characterization of organic anion transporter regulation, glutathione metabolism and bile formation in the obese Zucker rat. J. Hepatol..

[12] Canet M.J., Cherrington N.J. (2014). Drug disposition alterations in liver disease: Extrahepatic effects in cholestasis and nonalcoholic steatohepatitis. Expert Opin. Drug Metab. Toxicol..

[13] Zhang L., Xu P., Cheng Y., Wang P., Ma X., Liu M., Wang X., Xu F. (2019). Diet-induced obese alters the expression and function of hepatic drug-metabolizing enzymes and transporters in rats. Biochem. Pharmacol..

[14] Vilgrain V., Van Beers B.E., Pastor C.M. (2016). Insights into the diagnosis of hepatocellular carcinomas with hepatobiliary MRI. J. Hepatol..

[15] Planchamp C., Hadengue A., Stieger B., Bourquin J., Vonlaufen A., Frossard J.L., Quadri R., Becker C.D., Pastor C.M. (2007). Function of both sinusoidal and canalicular transporters controls the concentration of organic anions within hepatocytes. Mol. Pharmacol..

[16] Millet P., Moulin M., Stieger B., Daali Y., Pastor C.M. (2011). How organic anions accumulate in hepatocytes lacking Mrp2: Evidence in rat liver. J. Pharmacol. Exp. Ther..

[17] Bonnaventure P., Cusin F., Pastor C.M. (2019). Hepatocyte concentrations of imaging compounds associated with transporter inhibition: Evidence in perfused rat livers. Drug Metab. Dispos..

[18] Pastor C.M., Morel D.R., Billiar T.R. (1998). Oxygen supply dependence of urea production in the isolated perfused rat liver. Am. J. Respir. Crit. Care Med..

[19] Blouin A., Bolender R.P., Weibel E.R. (1977). Distribution of organelles and membranes between hepatocytes and nonhepatocytes in the rat liver parenchyma. A stereological study. J. Cell Biol..

[20] Segmental Linear Regression (2021). GraphPad Software, La Jolla California, USA.

[21] One Phase Decay (2021). GraphPad Software, La Jolla California, USA.

[22] Marrone J., Danielli M., Gaspari C.I., Capiglioni A.M., Marinelli R.A. (2021). Aquaporin gene transfer for hepatocellular cholestasis. Biochimie.

[23] Marinelli R.A., Vore M., Javitt N.B. (2019). Hepatic bile formation: Canalicular osmolarity and paracellular and transcellular water flow. J. Pharmacol. Exp. Ther..

[24] Ito K., Koresawa T., Nakano K., Horie T. (2004). Mrp2 is involved in benzylpenicillin-induced choleresis. Am. J. Physiol. Gastrointest. Liver Physiol..

[25] Pizarro M., Balasubramaniyan N., Solís N., Solar A., Duarte I., Miquel J.F., Suchy F.J., Trauner M., Accatino L., Ananthanarayanan M. (2004). Bile secretory function in the obese Zucker rat: Evidence of cholestasis and altered canalicular transport function. Gut.

[26] Lickteig A.J., Fisher C.D., Augustine L.M., Aleksunes L.M., Besselsen D.G., Slitt A.L., Manautou J.E., Cherrington N.J. (2007). Efflux transporter expression and acetaminophen metabolite excretion are altered in rodent models of nonalcoholic fatty liver disease. Drug Metab. Dispos..

